# Immobilisation of arsenic and simultaneous degradation of polycyclic aromatic hydrocarbons in soil in situ by modified electrooxidation

**DOI:** 10.1007/s11356-024-35878-z

**Published:** 2025-01-14

**Authors:** Jurate Kumpiene, Mariusz Gusiatin, Tanise Yang, Kim Johansson, Ivan Carabante

**Affiliations:** 1 Waste Science and Technology, Luleå University of Technology, Luleå, Sweden; 2 Department of Environmental Biotechnology, Faculty of Geoengineering, University of Warmia and Mazury in Olsztyn, Olsztyn, Poland

**Keywords:** In situ remediation, Electrokinetics, Creosote, Wood impregnation, PAH

## Abstract

Improper management of wood impregnation chemicals and treated wood has led to soil contamination at many wood treatment sites, particularly with toxic substances like creosote oil and chromated copper arsenate (CCA). The simultaneous presence of these pollutants complicates the choice of soil remediation technologies, especially if they are to be applied in situ. In this laboratory study, we attempted to immobilise arsenic (As) and simultaneously degrade polycyclic aromatic hydrocarbons (PAHs) (constituents of creosote oil) by applying a modified electrochemical oxidation method. The supply of iron (Fe) amendments in contaminated soil was done using corroding Fe electrodes as an Fe source and applying an alternating polarity electrical current. Soil with a large fraction of organic matter (25%) and containing 505 mg kg^−1^ As and 5160 mg kg^−1^ 16-PAHs was placed in Plexiglas cells equipped with porewater samplers and an iron electrode pair connected to a power supply unit. The porewater and percolating solution were periodically sampled and analysed over an 8-week period. The modified electrochemical soil treatment led to a decrease in the total concentration of 16-PAHs in soil by 56–68%. The amount of poorly crystalline Fe oxides in the soil substantially increased, especially close to the electrodes, enabling 76–89% of As to be bound to this most reactive Fe fraction. Nevertheless, over 10% of soil As remained in the most soluble and available fraction (exchangeable), most likely due to the decline in soil redox potential over time. This study suggests that electrochemical oxidation of organic soil with mixed contaminants could be used for in situ soil remediation but needs further improvement to achieve more efficient As immobilisation.

**Supplementary Information:**

The online version contains supplementary material available at 10.1007/s11356-024-35878-z.

## Introduction

Wood treatment has been carried out for many decades worldwide. However, improper management of treated materials and chemicals has resulted in soil contamination with various substances, requiring remedial actions. For example, creosote, chromated copper arsenate (CCA) and pentachlorophenol (PCP) used to be the main chemicals applied in the wood-preserving industry (Ottosen et al. 2002) but are now banned in many countries. Sites contaminated with these organic and inorganic compounds may pose risks to human health and the environment.

Among inorganic compounds, arsenic (As) is of particular concern due to its toxicity and mobility. In CCA-contaminated areas, As concentrations can vary by several orders of magnitude, from tens to thousands of mg kg^−1^ (Cao and Ma 2004; Frick et al. 2019; Kumpiene et al. 2006, 2023). Concentrations of Cr and Cu may also be high. In sites where creosote was used, high concentrations of polycyclic aromatic hydrocarbons (PAHs), constituents of creosote oil, have been found in soil and groundwater (Zapf-Gilje et al. 2001; Simpanen et al. 2016). At such sites, it may be difficult to select suitable soil remediation methods, especially ones that can be applied in situ for both types of contaminants, i.e. organic substances and inorganic elements.

Bioremediation, including phytotechnologies, are often preferred for in situ soil remediation. However, prior to using biotechnologies, including the utilisation of plants for remediation purposes, soil properties often need to be improved. This is usually achieved using soil amendments that reduce the contaminant toxicity and bioavailability (Kumpiene et al. 2008; Bolan et al. 2014). Metallic iron and iron-based amendments have been shown to be particularly effective for immobilisation of both As and co-occurring metals in soil (Cundy et al. 2008; Mench et al. 2010; Komarek et al. 2013). Corrosion of metallic iron added to soils produces poorly crystalline Fe(III) (oxyhydro)oxide minerals (mainly ferrihydrite), which react with dissolved ions and reduce their mobility (Kumpiene et al. 2006). Arsenic exhibits significant physical and chemical interactions with Fe(III) (oxyhydro)oxide minerals by the formation of inner-sphere complexes with surface hydroxyl groups of Fe oxides or by co-precipitation with Fe oxides during their formation and incorporation into the mineral structure (Fendorf et al. 1997; Voegelin et al. 2007).

One way of adding Fe to soil is by first excavating the soil, mixing it well with Fe amendments and then returning it to the original site (Okkenhaug et al. 2016; Nielsen et al. 2016). Spreading amendments on topsoil and ploughing can also be performed (Warren et al. 2003; Friesl-Hanl et al. 2009). However, if the contaminants are deeper than the ploughing depth or the soil is silty/clayey, mixing in amendments may be very difficult.

In situ soil amendment with iron has been tested by injection of nanoscale zerovalent iron (nZVI). However, aggregation of the soil particles and clogging of soil pores were observed to limit the nZVI distribution throughout soil profiles, especially in loamy or clayey soils (Phenrat et al. 2007; Yang et al. 2007). An attempt to increase the transport distance of nZVI in low permeability soils has been made by applying an external electric field to induce electrokinetic (EK)-mediated migration, with some positive results (Yang et al. 2007; Reddy et al. 2011; Fan et al. 2013). Nevertheless, application of a direct current (DC) caused strong acidification at the anode (pH 2.5–3), and alkalinisation at the cathode (pH 10–12). In addition, the problem of nanoparticle aggregation near the injection point remained unresolved (Gomes et al. 2013).

Electrokinetic remediation techniques were initially developed not to immobilise inorganic contaminants but to remove metals from soils, sludges and sediments (Virkutyte et al. 2002; FRTR 2024; Hamdi et al. 2025). In this process, a low-voltage DC is applied through inert electrodes to create a voltage gradient and electric field that induces the transport of metal ions towards oppositely charged electrodes and subsequent removal through various processes (e.g. Hassan and Mohamedelhassan 2012; Xie et al. 2021;). Later, the application of EK was expanded for the treatment of chlorinated and non-chlorinated volatile and semi-volatile organic compounds by facilitating the distribution of various amendments needed to initiate either chemical oxidation or reductive dichlorination of contaminants (FRTR 2024). Recently, the technique has been extended for the removal of a broad range of organic compounds, including petroleum hydrocarbons (diesel fuel, gasoline, lubricating oils, kerosene), PAHs and dense non-aqueous phase liquids (da Silva et al. 2017; Jayalakshmamma et al. 2021; Lim et al. 2016; Hamdi et al. 2025).

Surfactants are often used to increase the desorption and solubility of organic compounds, which then move towards electrodes through the aqueous-phase phenomena of electroosmosis and electromigration (Saichek and Reddy 2005). Such methods are referred to as electrokinetically enhanced in situ flushing (Hamdi et al. 2025). But the considerable potential of EK soil remediation lies its ability to degrade organic compounds directly within the soil, rather than merely desorbing and transporting them for subsequent removal (Proietto et al. 2023, 2024). Studies have shown that even at very low electric field strength (0.15 V cm^−1^), organic contaminants (e.g. phenols) can be simultaneously desorbed, mobilised and degraded in situ (Proietto et al. 2023).

The removal of metals applying the traditional EK also requires their solubilisation, e.g. by the addition of acids or complexing agents (Ahmed et al. 2022; Azhar et al. 2022). This is one of the main limitations of the traditional EK technique for remediation of metal contaminated soil because strong soil acidification is needed to prevent metals reaching zones of basic conditions and adsorbing to soil particles or precipitating in soil as e.g. (oxy)hydroxides (Virkutyte et al. 2002). However, this drawback has also been recently harnessed to deliberately precipitate inorganic contaminants in soil (Kumpiene et al. 2023; Purkis et al. 2023). Furthermore, corroding Fe electrodes can be used as a source of Fe particles to immobilise As and metals in contaminated soil, overcoming the high costs associated with using nZVI injection. Thus, it may be possible to substantially increase the method’s ability to treat mixed contaminants in situ by applying an electrical current to electrochemically disperse Fe to immobilise As in soil and concurrently degrade organic compounds.

The aim of this study, thus, was to apply a single method of electrooxidation in a novel way to simultaneously immobilise As in situ and degrade PAH in co-contaminated soil and evaluate the method by (i) analysing the supply of Fe amendments to soil from corroding electrodes operated with a low voltage electric current, (ii) evaluating Fe dispersion through soil and consequent immobilisation of As, and (iii) evaluating the extent of simultaneous PAH degradation in soil in laboratory settings.

## Materials and methods

### Soil

Soil contaminated with PAHs and As was collected at a former wood impregnation site in Limmared, south Sweden (57.54209°N, 13.34745°E) from a depth of 0–2 m using an excavator. At the site, creosote was used as an impregnant of railway sleepers, which was later replaced by CCA. Over 200 kg of soil was transported to the laboratory at LTU, where it was homogenised, subdivided and used in several experiments.

### Electrokinetic experiments

**Fig. 1:**
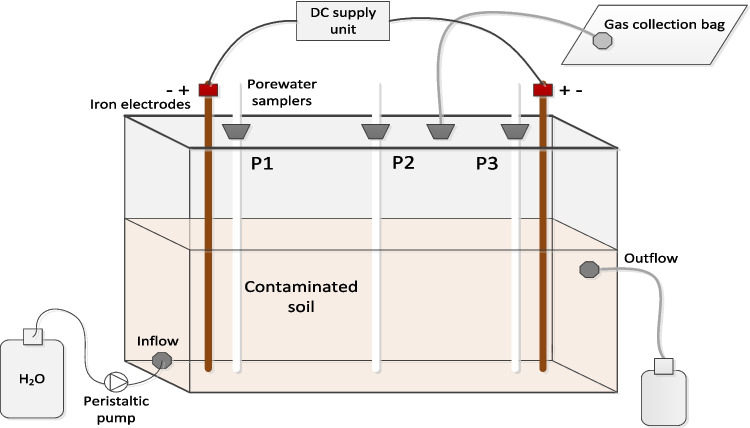
Experimental setup used for the electrokinetic remediation of PAH-As contaminated soil

Two identical laboratory cells, i.e. two replicates (Plexiglas *L* = 300 mm, *W* = 100 mm, *H* = 200 mm, 6 L), were each filled with 4 kg of contaminated soil and equipped with an iron electrode pair (Ø 5 mm, *L* = 270 mm) (Fig. 1). The electrodes were made from reinforcement iron rods. The rods were cleaned with 1 M HNO_3_ solution and distilled water prior to vertically inserting them through the soil to the bottom of the cells and connecting them to a power unit (DC Power Supply E3612A, Agilent). The electrical potential during the first 6 weeks of the experiment (43 days) was fixed to give an electric field strength or voltage potential of 2.4 V cm^−1^, which was afterwards reduced to 0.64 V cm^−1^. The current was let to float and was varying during the experiment between 40 and 290 mA. The current direction was alternated daily to allow the electrodes to function both as cathodes and as anodes and corrode equally.

Porewater samplers (MacroRhizon soil moisture samplers, *L* = 9 cm, Ø 4.5 mm; Niekamp, the Netherlands) were installed next to the electrodes and in the centre of the cells for weekly sampling and analyses. To collect sufficient volumes of leachate needed for the PAH analysis, the cells were run for 8 weeks in a flow-through mode using distilled water at a flow rate of 11 ml min^−1^ generated by a peristaltic pump (Watson Marlow 520S). The outflow solution was collected in glass bottles covered with aluminium foil to prevent exposure to light. Small volume (ca. 10 mL) subsamples of the leachate were taken at the same frequency as porewater and filtered through a 0.45-μm nitrocellulose membrane filter before analysis. Cumulative leachate samples were collected on eight occasions for PAH analysis.

During the experiment, the cells were tightly covered with a lid to collect gases in the air space above the soil. Tedlar bags were connected to avoid overpressure in the cells.

At the end of the experiment, soil samples were collected next to the electrodes and in the middle of the cell (between the electrodes), and sequential extraction was performed to identify changes in the Fe and As distribution between the operationally defined soil fractions as affected by the treatment. For comparison, a control soil sample of ca 3 kg was stored in a closed container under the same temperature conditions and field moisture capacity without any treatment and analysed at the end of the experiment for As and Fe distribution among soil fractions in the same way as the soils in the treated cells.

### Sequential extraction

The detailed procedure for sequential extraction is described by Kumpiene et al. (2012). The following six fractions were extracted: (i) exchangeable fraction, extracted with 1 M ammonium acetate at pH 4.5; (ii) poorly crystalline Fe(III)-(oxyhydro)oxide fraction, extracted with 0.2 M ammonium oxalate at pH 3.0; (iii) crystalline Fe(III) (oxyhydro)oxide fraction, extracted with 0.2 M ammonium oxalate at pH 3.0 and 80 °C; (iv) Fe/Mn oxide fraction, extracted with 0.04 M hydroxylamine hydrochloride in 25% (v/v) acetic acid at pH 2 and 96 °C; (v) organic matter and secondary sulphide fraction, extracted with 35% hydrogen peroxide at 85 °C; and (vi) residual fraction, extracted with aqua regia (HNO_3_:HCl, 1:3 v/v) at 195 °C in a microwave digester (Microwave Reaction System, Multiwave PRO, Anton Paar). All extracts were filtered through 0.45 μm cellulose acetate syringe filters and stored at 4 °C prior to analysis by inductively coupled plasma optical emission spectrometry (ICP-OES, Optima 8300, PerkinElmer). The total concentration of elements was calculated as the sum of all fractions.

### Analyses

The pH, redox potential and electrical conductivity (EC) were immediately measured after each sampling of porewater and outflow solution. The As and Fe concentrations in the solutions were analysed using ICP-OES. The pH, redox potential and EC of solid soil samples were measured in soil-distilled water suspensions (1:2.5 v:v) after 2 h equilibration time. The organic matter content was measured as loss on ignition at 550 °C.

The outflow water was sent to the accredited laboratory ALS Scandinavia on eight occasions for analysis of 16-PAHs following the USEPA method 8310 (USEPA 1986). The untreated (control) soil and soil samples collected from the cells at the end of the experiment were sent for 16-PAH analysis as well.

Dissolved organic carbon (DOC) in the outflow solution was measured with a total organic carbon analyser (TOC VCPH/CPN, Shimadzu Corporation) according to European standard EN 1484 (CEN 1998).

Gases collected in the air space above the soil were analysed for total volatile organic compounds (VOC) using a photoionisation detector (PID) (MiniRAE 3000, Honeywell Analytics) once a week. The instrument was calibrated using 100 ppm isobutylene calibration gas. The measurements were implemented by pumping the gases directly from the overhead space of the cells through the detector until a steady reading was achieved (ca 10 s). The composition of gases that included concentrations of carbon dioxide, oxygen, nitrogen, methane and hydrogen sulphide in the air space were measured once a month using gas chromatography (GC) (PerkinElmer, Clarus 500). The gas samples were extracted from the cells using a syringe and injected into the sample vials. Measurements were done in triplicates and average values were calculated.

The *Statgraphics 19* software was used for analysis of variance (ANOVA) and regression analysis. Fisher's least significant difference (LSD) procedure (*p* < 0.05) was used to discriminate among the sample means.

## Results and discussion

### Soil properties

The studied soil was rich in organic matter (peat) and contained high concentrations of both As and PAHs, which substantially exceeded the Swedish guideline values for soil with less sensitive land use (Swedish EPA 2022; Table 1). Although the use of creosote and CCA ceased several decades ago, constituents of both these chemicals were still present in the soil. However, Cu and Cr, which are also components of CCA, were found in relatively low concentrations and did not exceed the guideline values (Table 1).

**Table 1:** Main properties of the contaminated soil used in the electrokinetic experiment

	pH	EC	Redox potential	OM^a^	As	Cr	Cu	Fe	PAHs			
									L	M	H	Sum16
	-	mS cm^−1^	mV	%	mg kg^−1^				mg kg^−1^			
Contaminated soil	5.3 ± 0.0	142 ± 4	292 ± 6	25 ± 7	502 ± 15	66 ± 5	63 ± 2	24,482 ± 2145	841	3380	940	5160
Guideline values^b^					25	150	200	-	15	20	10	-

^a^Organic matter, measured as loss on ignition at 550 °C

^b^Swedish guideline values for contaminated soil with less sensitive land use (Swedish EPA, 2022)

The soil contained a high initial concentration of 16-PAHs (5160 mg kg^−1^). After dividing PAHs into three groups based on molecular size, PAHs with medium molecular size (4-rings, PAH-M) were found to be dominant, as previously observed at other sites where impregnation of railroad sleepers with creosote oil occurred (Simpanen et al. 2016). All groups of PAHs exceeded the Swedish guideline values for PAHs in soil with less sensitive land use (Table 1).

### Development of electrical conductivity, pH and redox potential in soil solution

**Fig. 2:**
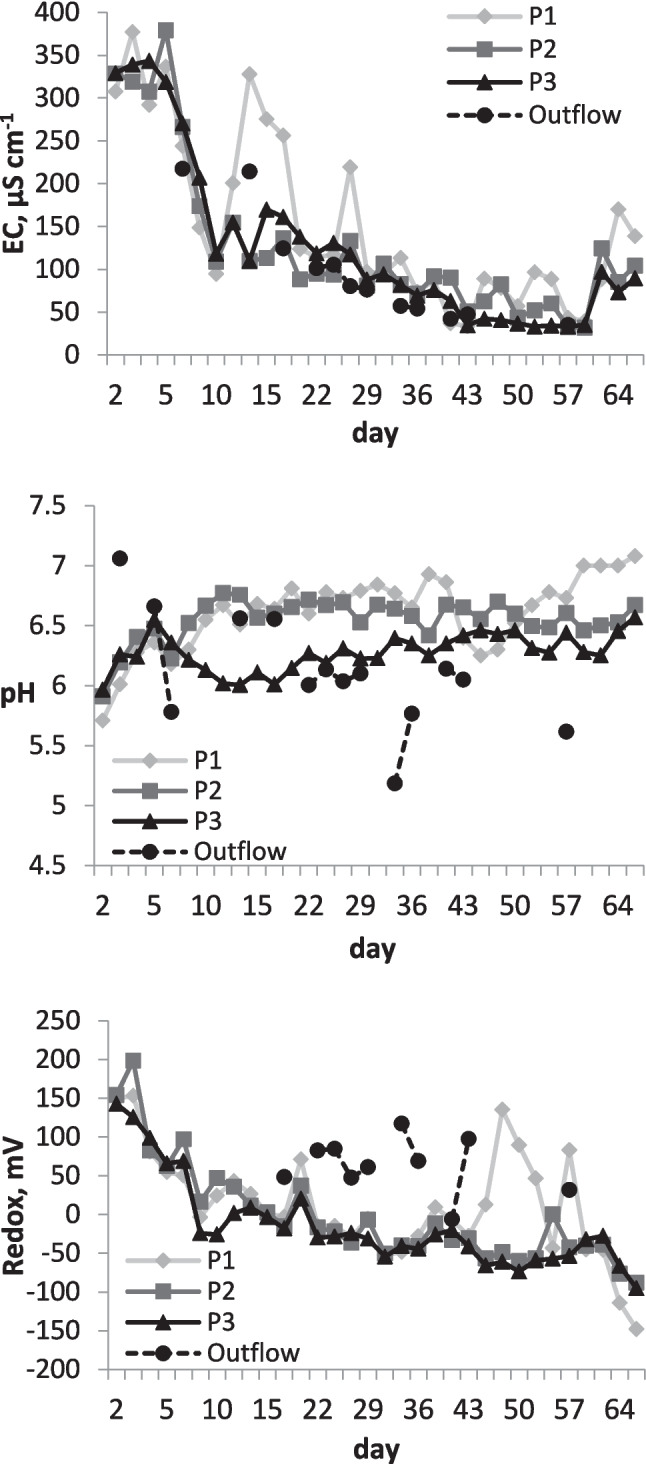
Average pH, redox potential and electrical conductivity (EC) of porewater collected at three sampling points (P1, P2, P3) and the outflow solution of both cells

Figure 2 shows the variation in electrical conductivity (EC), pH and redox potential in cells containing contaminated soil. The EC decreased during the 2-month experiment, which is a common phenomenon observed in electrokinetic soil remediation for the following reasons. When a DC electric field is applied to soil, exchangeable ions are initially desorbed from the soil particles into the porewater, increasing the EC. However, as the desorbed and dissolved ions migrate through the soil (electromigration) or are transported together with soil porewater (electroosmosis), they eventually become readsorbed or precipitated in soil pores, and the conductivity gradually decreases, which in turn can cause a continuous decrease in the current being carried by the ions (Viadero et al. 1998; Kim et al. 2012). The decreased value of EC indicate that the concentration of dissolved ions in the soil solution decreased, consistent with the aim of the soil treatment.

The pH slightly increased during the test from 5.7–6.0 to 6.6–7.0 in the three porewater sampling points, whereas redox values decreased from 150 mV to − 90 to − 150 mV. Some deviating pH and redox values were observed in the outflow solution (Fig. 2). Often, pH and redox potential are confounding factors and an increase in pH is accompanied by a decrease in redox potential (Han et al. 2019; Ma et al. 2022). Changing the current direction and electrode polarity was expected to counteract the variations in pH and redox conditions at the electrodes. Strong acidification at the anode, reaching as low as pH 2, and strong alkalinisation at the cathode, reaching pH 12, usually develop when a direct current electric field is used (Yeung 2011). Such conditions are preferred when the aim is to move ions towards electrodes for subsequent removal. However, in the present study, relatively stable pH-redox conditions were preferred to preserve the soil properties. Thus, alternating the current helped to keep the pH relatively stable throughout the experiment, although a slight increase by up to one pH unit was observed after ca. 1 month, indicating that a slight surplus of hydroxyl ions was formed in the soil. However, the magnitude of the decrease in redox potential was unexpected and may have occurred because of a lack of sufficient inflow of oxygen as the cells were tightly closed. In addition, the soil was water saturated and rich in organic matter. Therefore, the conditions were unfavourable for maintaining soil oxygenation. Furthermore, this result may indicate that an excess of free electrons was formed, which could have contributed to reducing conditions in the soil. Both, soil alkalinisation and redox drop, are unfavourable for the immobilisation of As. To counteract this, the electric field strength was decreased from 2.4 V cm^−1^ to 0.64 V cm^−1^ after 43 days by lowering the voltage potential of the electrodes. Such a change has also been expected to reduce ion migration in soil (Sun et al. 2023). After altering the electric field strength, a sharp increase in the redox potential at the first electrode and some stabilisation in the rest of the cell were observed, but the effect did not persist for more than 1 month, and then, the redox potential decreased again by the end of the experiment (Fig. 2).

### Stabilisation of As

**Fig. 3:**
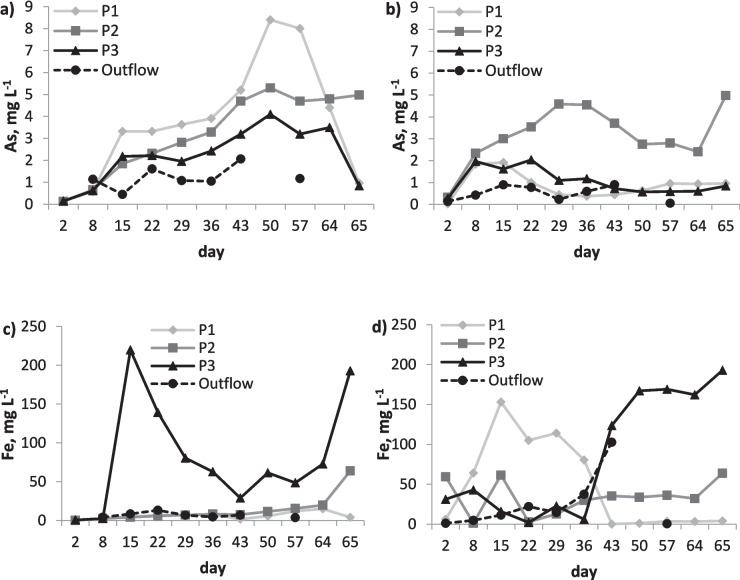
Porewater and outflow solution concentrations of arsenic in **a** cell 1 and **b** cell 2 and iron in **c** cell 1 and **d** cell 2

The decrease in redox potential had a negative effect on the stability of As in soil. Arsenic is a redox-sensitive element, and its mobility usually increases with decreasing redox potential, as the main As-bearing phases in soil—Fe oxides—also dissolve, releasing bound As (Signes-Pastor et al. 2007; Han et al. 2019; Zhang et al. 2023). Indeed, the As concentration in the porewater gradually increased over time and then decreased after the decrease in electric field strength on day 43 (Fig. 3a). Nevertheless, the decrease in dissolved As was only observed at the electrodes, whereas in the centre of the cell (sampling point P2), the dissolved As concentration remained high. This may have been influenced by various factors, such as the soil heterogeneity and variations in the electric field distribution.

Electrochemical reactions in soil are extremely complex and soil specific (Yeung and Gu 2011). Although the two cells were identical and filled with well-homogenised soil, their results varied, which may have been due to differences in the contaminant distribution in subsamples. Also, creosote oil contaminated organic soil was  difficult to homogenise, and it is possible that there was a difference in soil texture between the soils packed in the cells, which might have affected the electric field distribution. This in turn may have caused differences in ion migration and extent of chemical reactions in the two cells. For this reason, the results for dissolved As and Fe in soil are presented for each cell separately (Fig. 3).

The dissolved Fe concentration was highest at sampling point P3 (Fig. 3c, d), which was the last point in the cells in the direction of water flow. It is possible that Fe that dissolved in the soil was transported to this point, causing an accumulation of Fe there. At the same time, the concentration of As was the lowest at this point (Fig. 3a, b), suggesting that some of the Fe reacted with As, causing its immobilisation.

Migration of dissolved Fe towards a cathode electrolyte was also observed by Zhang et al. (2023), who studied the electrokinetic removal of As in paddy soil. In addition, the authors observed an increase in the content of amorphous Fe in the soil, possibly due to the secondary precipitation of dissolved Fe. Some migration of Fe in sandy soil was observed in field experiments using Fe electrodes and a low-voltage electric field to stabilise As-contaminated soil (Kumpiene et al. 2023). Corrosion of the Fe electrodes and the highest Fe concentration (mainly as poorly crystalline Fe oxides) were detected near the electrodes.

**Fig. 4:**
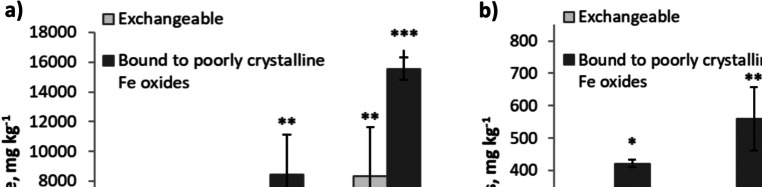
Concentrations of **a** iron and **b** arsenic in exchangeable and poorly crystalline Fe oxide fractions in treated soil at an Fe electrode, middle of the cell and in the control soil. The error bars indicate standard deviations of the means (*n* = 3–6). Different numbers of asterisks show statistically significant differences between the sample means at 95% confidence level

In the present study, an increase in Fe concentration (mainly Fe in exchangeable and poorly crystalline fractions) was also identified in treated soil compared with untreated soil, as evaluated by sequential extraction (Fig. 4a). This indicates that Fe corrosion occurred (also confirmed by thinning of the Fe electrodes) and Fe compounds slowly spread through the soil. No significant changes were observed in any other Fe fraction as a result of the soil treatment.

The poorly crystalline Fe oxide fraction was the second most abundant in the soil (23–36% of soil Fe) after the residual fraction (41–68% of soil Fe). A change in the poorly crystalline Fe oxide fraction is one of the most relevant indicators of soil treatment success as these oxides are the most reactive towards the soil contaminants. The adsorption capacity of poorly crystalline Fe oxides (ferrihydrite, lepidocrocite, goethite) is greater than that of crystalline Fe oxides, such as hematite (Kumpiene et al. 2012; Wang et al. 2018) due to their high specific surface area and abundance of functional groups. An increase in the amount of reactive Fe oxides (from 23% in the control soil to 29% in the middle of the treatment cell and 36% at the electrodes) indicates that more Fe oxide surfaces became available for As immobilisation.

The concentration of As bound to poorly crystalline Fe oxides increased significantly in the treated soil, especially at the electrodes (from 421 mg kg^−1^ in control to 674 mg kg^−1^ in treated soil; Fig. 4b). The As concentration bound to this fraction was dominant, representing 81–87% of all soil As. Together with the exchangeable As, these two fractions made up 95–97% of all soil As. Nevertheless, a substantial concentration of As remained in the exchangeable fraction (10–16% of soil As) that did not react with Fe oxides. Therefore, despite the considerable concentration of As in the poorly crystalline Fe oxide fraction, the immobilisation of soluble As was not very successful, most likely due to the low soil redox potential and incomplete precipitation of dissolved Fe. Under aerobic conditions (+ 400–0 mV), As can co-precipitate or become immobilised by sorption to newly formed hydrated iron oxides, but the solubility of As and Fe starts to increase at redox values of − 50 to − 150 mV (Signes-Pastor et al. 2007), which was evident in cell 1 for As (Fig. 3a) and both cells for Fe (Fig. 3c, d).

**Fig. 5:**
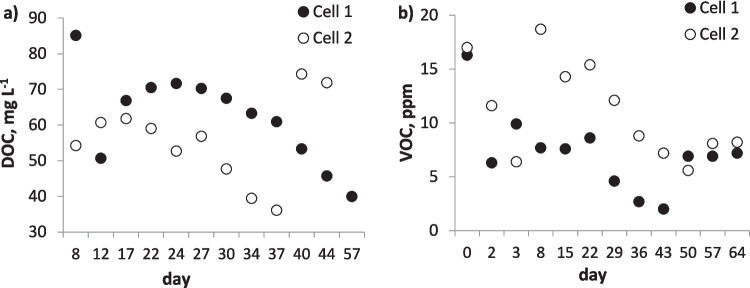
Concentrations of **a** dissolved organic carbon (DOC) in the outflow solution and **b** volatile organic compounds (VOCs) in the air of cells 1 and 2

In the present study, the prevalence of dissolved organic carbon (DOC) in the soil solution (Fig. 5) might also have affected As mobility. DOC can either strongly interact with Fe oxides, competing with As for binding sites (Kim et al. 2015; Sharma et al. 2010; Aftabtalab et al. 2022), or can inhibit the secondary precipitation of Fe(III) (hydro)oxides with As, thereby reducing its immobilisation (Wu et al. 2019). In addition, the low redox potential measured in the soil may indicate that DOC itself served as an electron donor (Suda and Makino 2016), contributing to the surplus of electrons in the soil that already contained insufficient electron acceptors. Therefore, further studies are needed to develop electrochemical processes that allow stimulation of soil oxygenation or addition of supplementary electron acceptors.

### Degradation of PAHs

**Fig. 6:**
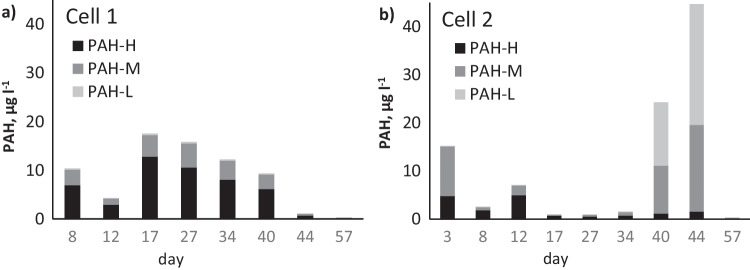
Concentrations of low (L), medium (M) and high (H) molecular weight PAHs in the outflow solution from **a** cell 1 and **b** cell 2

The concentrations of 16-PAHs in outflow water, subdivided into low (PAH-L), medium (PAH-M) and high (PAH-H) molecular weight PAHs, are presented in Fig. 6. Concentrations of each of the 16-PAHs in solution are given in the Supplementary Material, Table S1. Leached concentrations differed somewhat between the cells, and in cell 2 increased significantly at the end of the experiment, especially for PAH-L (Fig. 6).

It is possible that this increase was due to the release of PAHs from soil into the solution, which is preferred, because PAHs in solution are more easily available for degradation than those bound to soil. The desorption of PAHs is expected to occur in electrokinetically treated soils, leading to an initial decrease and then temporal increase in dissolved PAHs (Ammami et al. 2014), followed by a decrease again as PAHs degrade. Indeed, the final dissolved PAH concentrations measured on day 57 had decreased to the lowest values in both cells, i.e. 0.33 µg L^−1^ in cell 1 and 0.32 µg L^−1^ in cell 2.

Most PAHs have a high affinity for soil organic matter and low solubility in water, which decreases with increasing number of rings in the PAH molecules (Patel et al. 2020). Organic pollutants, such as oils and PAHs, are electrically neutral. Thus, their movement in soil is affected by the electrical current mainly through electroosmotic flow (Alcántara et al. 2012) and increases at higher voltage (Yang and Lee 2009). Since the aim of the electrochemical soil treatment was to increase the oxidative degradation of these compounds rather than increase the migration of PAHs towards the electrodes, replenishment of PAHs in the soil solution through desorption from the soil was needed. One way to achieve this is to increase the concentration of DOC in the soil solution. Because electrooxidation is not compound-specific, soil organic matter may undergo oxidation, which increases the amount of DOC in soil. It is known that the presence of dissolved humic matter increases the aqueous solubility of PAHs, especially compounds with low molecular weight (2 ring PAHs). Lassen et al. (1997) observed that addition of 25 and 50 mg L^−1^ humic acids to the water phase increased the solubility of phenanthrene by 17 and 42%, respectively, compared to pure water. Organic matter–rich soils, such as the peat studied here, are expected to supply DOC, and hence increase PAH desorption from the soil surface. The concentration of DOC measured in the outflow solution followed a similar trend to that of dissolved PAHs; i.e., DOC increased slightly over time but then decreased (Fig. 5a). It is likely that the dissolution of PAHs and soil organic matter was enhanced by the electrochemical treatment. However, more detailed studies are needed to determine whether there was any causation between these factors.

As the treatment proceeded, the dissolved PAHs were expected to degrade, resulting in a decrease in PAH concentrations in both the soil and solution. The initial concentration of 16-PAHs in the untreated soil was 5160 mg kg^−1^ (Table 1). At the end of the experiment, the PAH concentration had decreased in cell 1 to 2280 mg kg^−1^ and in cell 2 to 1640 mg kg^−1^. Thus, the increase in dissolved PAHs in cell 2 at the end of the experiment indicates that the replenishment of PAHs from the soil to solution was accompanied by increased overall degradation of PAHs in the soil.

The composition of the 16-PAHs in the soil of cell 1 at the end of the experiment was PAH-L = 141 mg kg^−1^, PAH-M = 1780 mg kg^−1^ and PAH-H = 351 mg kg^−1^, whereas in cell 2, the corresponding values were PAH-L = 131 mg kg^−1^, PAH-M = 1230 mg kg^−1^ and PAH-H = 279 mg kg^−1^. Thus, there was a significant decrease in all PAH fractions following the electrochemical treatment compared with the untreated soil (Table 1). Furthermore, although all PAH fractions degraded simultaneously, the PAH-L fraction decreased the most (on average by 84%), followed by PAH-H (by 66%) and then PAH-M (by 55%). These results imply that the electrochemical treatment was able to degrade all PAHs in soil within a relatively short period of time.

Degradation of PAHs in soil subjected to an electric field is a complex process that can occur directly by the transfer of electrons from the organic molecule to anode or indirectly by splitting water molecules induced by the electric field and formation of reactive oxygen species that react with organic pollutants (Ajab et al. 2020). Moreover, the rapid degradation of PAHs may have been facilitated by alternating the polarity of the electrodes during the electrochemical treatment. Alternating the polarity has previously been shown to increase PAH degradation compared to using a constant one-directional direct current flow (Li et al. 2012). By periodically reversing the current flow, the movement direction of water, colloids and ions frequently changes, increasing the contact and interaction opportunities between soil microorganisms, nutrients and contaminants (Li et al. 2012). Niqui‐Arroyo and Ortega‐Calvo (2007) observed that use of periodic changes in polarity and a pulsing current increased the removal of total PAHs by 16% and that of larger molecular weight PAHs, e.g. benzo[a]pyrene, by up to 30%.

### Release of gases during electrochemical soil treatment

The generation of total volatile organic compounds (VOCs) indicates the presence of fractions of organic matter that can easily volatilise and spread from the soil through air. The measured values were relatively low and after some variation decreased from initial values of 16–17 ppm to 7–8 ppm by the end of the experiment (Fig. 5b). Thus, the volatilisation of organic contaminants during electrochemical treatment was deemed negligible.

Analysis of the gas composition in the air space of the two cells showed that the carbon dioxide concentration increased, whereas the oxygen concentration decreased over time, indicating degradation of organic matter in the soil, including PAHs, and the release of CO_2_ as the final degradation product (Table 2). The concentrations of methane and hydrogen sulphide in the cells were below detectable levels.

**Table 2:** Percentages of carbon dioxide, nitrogen and oxygen in gases collected in the air space above the soil

	Carbon dioxide	Oxygen	Nitrogen
	%		
Cell 1			
Month 1	0.27	20.30	79.43
Month 2	2.15	15.33	82.53
Cell 2			
Month 1	2.95	15.53	81.52
Month 2	2.98	14.65	82.36

Part of the CO_2_ may have been produced by microorganisms that contributed to the degradation of PAHs. The activity and metabolism of soil microorganisms are usually stimulated by low intensity electrical current, which can occur directly by the transfer of electrons from the electrode to the bacteria or indirectly by the transfer of electrons through the hydrolysis of water (Niqui‐Arroyo and Ortega‐Calvo 2007; Mena et al. 2014; Camacho, 2021). A pilot-scale study by Li et al. (2020) on the removal of PAHs from contaminated soil (total concentration of PAHs of 5636 mg kg^−1^) showed that electrochemically assisted bioremediation improved PAH degradation, especially of larger PAHs (> 3 rings). The degradation of PAHs with four and six rings after 182 days reached 69% and 66%, respectively, and was about 40% and 22% higher than the degradation of these PAHs with bioremediation alone (Li et al. 2020).

## Conclusions

The electrochemical oxidation of organic soil from a former wood impregnation site using alternating polarity low voltage DC current and Fe electrodes resulted in a decrease of the total 16-PAH concentration in soil by 56–68% during a 2-month laboratory experiment. All PAH fractions in the soil and solution degraded simultaneously, but low molecular weight PAHs in soil decreased the most (on average by 84%), followed by high molecular weight PAH fractions (by 66%) and then medium molecular weight PAHs (by 55%).

Furthermore, the amount of poorly crystalline Fe oxides substantially increased, enabling 76–89% of As to be bound to this most reactive Fe fraction. The concentration of Fe and abundance of poorly crystalline Fe oxides were largest closest to the electrodes, mainly due to corrosion of the Fe electrodes. Nevertheless, over 10% of As was present in the most soluble and available fraction (exchangeable), which needs to be reduced before the method can be considered successful for As immobilisation. The decrease in the soil redox potential over time was the most likely reason that As was still found in the soil solution, despite the increased availability of Fe oxides.

In summary, this study suggests that the modified electrochemical oxidation of organic soil with mixed contaminants could be used for in situ soil remediation but needs further improvement focusing on redox control and more complete immobilisation of As.

## Supplementary Information

Below is the link to the electronic supplementary material.

Supplementary file1 (DOCX 69 KB)

## Data Availability

The authors declare that the data supporting the findings of this study are available within the paper and its supplementary information files. Should any raw data files be needed in another format, they are available from the corresponding author upon reasonable request.
