# Supplementary material for: Immobilisation of arsenic and simultaneous degradation of polycyclic aromatic hydrocarbons in soil in situ by modified electrooxidation
Source: Environ Sci Pollut Res Int. 2025 Jan 14;32(5):2656–67. doi: 10.1007/s11356-024-35878-z (PMC11802621; doi:10.1007/s11356-024-35878-z)
Supplement: Supplementary file 1 — Supplementary file1 (DOCX 69 KB) [file 11356_2024_35878_MOESM1_ESM.docx]

**Supplementary Material**

to article:

**Immobilisation of arsenic and simultaneous degradation of polycyclic aromatic hydrocarbons in soil *in situ* by modified electrooxidation**

Jurate KUMPIENE^1^ ^[[1]](#footnote-1)^, Mariusz GUSIATIN^2^, Tanise YANG^1^, Kim JOHANSSON^1^, Ivan CARABANTE^1^

^1^Waste Science and Technology, Luleå University of Technology, Sweden

^2^ Department of Environmental Biotechnology, Faculty of Geoengineering, University of Warmia and Mazury in Olsztyn, Poland

**Table S1**. Concentrations of 16-PAH in soil solution collected at the outflow of cells 1 and 2. < values below instrument detection limits.

| **Sampling day** | **8** | **12** | **17** | **27** | **34** | **40** | **44** | **57** |
| --- | --- | --- | --- | --- | --- | --- | --- | --- |
| **Cell 1** | µg L^-1^ | | | | | | | |
| naphthalene | <0.010 | <0.010 | <0.014 | <0.014 | <0.014 | <0.014 | <0.014 | <0.030 |
| acenaphthylene | 0.085 | 0.050 | 0.146 | 0.125 | 0.097 | 0.074 | 0.025 | 0.026 |
| acenaphthene | 0.127 | 0.047 | 0.174 | 0.165 | 0.138 | 0.121 | 0.023 | <0.010 |
| fluorene | <0.174 | <0.084 | 0.105 | 0.113 | 0.090 | <0.127 | <0.147 | <0.010 |
| phenanthrene | 0.207 | 0.074 | 0.331 | 0.357 | 0.297 | 0.230 | 0.036 | <0.010 |
| anthracene | 0.152 | 0.081 | 0.253 | 0.236 | 0.188 | 0.152 | 0.068 | 0.086 |
| fluoranthene | 1.640 | 0.652 | 2.140 | 2.220 | 1.840 | 1.510 | 0.149 | 0.048 |
| pyrene | 1.200 | 0.489 | 1.600 | 2.020 | 1.490 | 1.110 | 0.118 | 0.035 |
| benz[a]anthracene | 0.611 | 0.231 | 0.920 | 1.010 | 0.867 | 0.678 | 0.037 | 0.012 |
| chrysene | 0.653 | 0.260 | 0.833 | 0.897 | 0.562 | 0.460 | 0.038 | <0.010 |
| benzo[b]fluoranthene | 2.570 | 1.100 | 5.030 | 4.030 | 3.120 | 2.390 | 0.284 | 0.051 |
| benzo[k]fluoranthene | 0.908 | 0.389 | 1.600 | 1.290 | 0.981 | 0.699 | 0.099 | 0.016 |
| benzo[a]pyrene | 1.420 | 0.594 | 2.490 | 1.910 | 1.420 | 1.090 | 0.134 | 0.018 |
| benzo[a,h]perylene | 0.127 | 0.056 | 0.318 | 0.239 | 0.184 | 0.133 | 0.018 | <0.010 |
| dibenz[g,h,i]anthracene | 0.237 | 0.104 | 0.633 | 0.474 | 0.378 | 0.280 | 0.040 | 0.018 |
| indeno[1,2,3-cd]pyrene | 0.421 | 0.182 | 1.030 | 0.741 | 0.586 | 0.439 | 0.060 | 0.022 |

**Continuation of Table S1.**

| **Sampling day** | **3** | **8** | **12** | **17** | **27** | **34** | **40** | **44** | **57** |
| --- | --- | --- | --- | --- | --- | --- | --- | --- | --- |
| **Cell 2** | µg L^-1^ | | | | | | | | |
| naphthalene | <0.010 | <0.010 | <0.010 | <0.014 | <0.014 | <0.014 | <0.014 | 0.326 | 0.014 |
| acenaphthylene | 0.075 | 0.061 | 0.076 | 0.028 | 0.018 | 0.023 | 0.132 | 0.335 | <0.010 |
| acenaphthene | 0.048 | 0.020 | 0.047 | <0.014 | 0.035 | 0.136 | 13.100 | 24.400 | <0.010 |
| fluorene | <0.361 | <0.266 | <0.172 | <0.138 | <0.239 | <0.295 | 3.880 | 7.940 | <0.010 |
| phenanthrene | 0.058 | 0.029 | 0.084 | 0.017 | 0.021 | <0.014 | 0.077 | 1.760 | 0.070 |
| anthracene | <0.098 | 0.060 | 0.097 | 0.030 | <0.038 | <0.042 | <0.098 | <0.279 | 0.019 |
| fluoranthene | 6.800 | 0.341 | 1.130 | 0.104 | 0.182 | 0.341 | 4.050 | 5.490 | 0.014 |
| pyrene | 3.430 | 0.190 | 0.701 | 0.073 | 0.119 | 0.359 | 1.940 | 2.840 | <0.010 |
| benz[a]anthracene | 1.580 | 0.470 | 0.861 | 0.054 | 0.085 | 0.182 | 0.333 | 0.484 | <0.010 |
| chrysene | 1.140 | 0.349 | 1.010 | 0.046 | 0.075 | 0.163 | 0.263 | 0.383 | 0.097 |
| benzo[b]fluoranthene | 0.955 | 0.494 | 1.490 | 0.282 | 0.182 | 0.180 | 0.245 | 0.333 | 0.030 |
| benzo[k]fluoranthene | 0.342 | 0.156 | 0.475 | 0.077 | 0.058 | 0.063 | 0.088 | 0.117 | 0.039 |
| benzo[a]pyrene | 0.488 | 0.241 | 0.708 | 0.122 | 0.080 | 0.075 | 0.115 | 0.171 | <0.010 |
| benzo[a,h]perylene | 0.044 | 0.019 | 0.071 | 0.019 | <0.014 | <0.014 | 0.015 | 0.015 | <0.010 |
| dibenz[g,h,i]anthracene | 0.091 | 0.044 | 0.135 | 0.042 | 0.024 | 0.021 | 0.036 | 0.032 | 0.014 |
| indeno[1,2,3-cd]pyrene | 0.152 | 0.073 | 0.224 | 0.065 | 0.041 | 0.035 | 0.056 | 0.055 | 0.297 |

**Table S2**. Concentrations (µg kg^-1^) and proportions (%) of individual PAH in untreated soil (control) and soil collected at the end of the experiment in cells 1 and 2.

| **PAH** | **Control** |  | **Cell 1** |  | **Cell 2** |  |
| --- | --- | --- | --- | --- | --- | --- |
|  | µg kg^-1^ | **%** | µg kg^-1^ | **%** | µg kg^-1^ | **%** |
| naphthalene | 234.0 | 4.5 | 4.6 | 0.2 | 2.6 | 0.2 |
| acenaphthylene | 14.9 | 0.3 | 3.9 | 0.2 | 3.0 | 0.2 |
| acenaphthene | 592.0 | 11.5 | 133.0 | 5.8 | 125.0 | 7.6 |
| fluorene | 476.0 | 9.2 | 131.0 | 5.8 | 80.3 | 4.9 |
| phenanthrene | 643.0 | 12.5 | 680.0 | 29.9 | 405.0 | 24.7 |
| anthracene | 138.0 | 2.7 | 94.3 | 4.1 | 50.7 | 3.1 |
| fluoranthene | 1060.0 | 20.6 | 560.0 | 24.6 | 443.0 | 27.0 |
| pyrene | 1060.0 | 20.6 | 319.0 | 14.0 | 251.0 | 15.3 |
| benz[a]anthracene | 377.0 | 7.3 | 110.0 | 4.8 | 85.6 | 5.2 |
| chrysene | 281.0 | 5.4 | 87.0 | 3.8 | 80.5 | 4.9 |
| benzo[b]fluoranthene | 136.0 | 2.6 | 59.8 | 2.6 | 45.4 | 2.8 |
| benzo[k]fluoranthene | 47.5 | 0.9 | 29.1 | 1.3 | 22.5 | 1.4 |
| benzo[a]pyrene | 65.2 | 1.3 | 29.8 | 1.3 | 20.7 | 1.3 |
| benzo[a,h]perylene | 6.2 | 0.1 | 6.1 | 0.3 | 4.2 | 0.3 |
| dibenz[g,h,i]anthracene | 12.3 | 0.2 | 11.5 | 0.5 | 7.7 | 0.5 |
| indeno[1,2,3-cd]pyrene | 14.8 | 0.3 | 17.3 | 0.8 | 12.4 | 0.8 |
| Sum | 5160 | 100 | 2276 | 100 | 1640 | 100 |

1. Corresponding author : Jurate Kumpiene, jurate.kumpiene@ltu.se [↑](#footnote-ref-1)
